# A Cluster-Randomized Controlled Trial Evaluating the Effectiveness and Cost-Effectiveness of Tobacco Cessation on Prescription in Swedish Primary Health Care: A Protocol of the Motivation 2 Quit (M2Q) Study

**DOI:** 10.2196/resprot.6180

**Published:** 2016-09-16

**Authors:** Anne Leppänen, Peter Lindgren, Carl Johan Sundberg, Max Petzold, Tanja Tomson

**Affiliations:** ^1^ Karolinska Institutet Department of Learning, Informatics, Management and Ethics Stockholm Sweden; ^2^ The Swedish Institute for Health Economics Lund Sweden; ^3^ Karolinska Institutet Department of Physiology and Pharmacology Stockholm Sweden; ^4^ University of Gothenburg Department of Public Health and Community Medicine Gothenburg Sweden

**Keywords:** tobacco use cessation, primary health care, vulnerable populations, randomized controlled trial, pragmatic clinical trial, cost-effectiveness analysis, Sweden

## Abstract

**Background:**

In Sweden, the prevalence of tobacco use is disproportionately high among socioeconomically disadvantaged groups. Previous research and clinical experience suggest that prescribed lifestyle interventions in the primary health care (PHC) setting such as Physical Activity on Prescription are effective in changing behavior. However, there is a lack of evidence for if and how such a prescription approach could be effectively transferred into the tobacco cessation context.

**Objective:**

The aim of this trial is to evaluate the effectiveness and cost-effectiveness of Tobacco Cessation on Prescription (TCP) compared to current practice for tobacco cessation targeting socioeconomically disadvantaged groups in the PHC setting in Sweden.

**Methods:**

The design is a pragmatic cluster-randomized controlled trial. The sample will consist of 928 daily tobacco users with Swedish social security numbers and permanent resident permits, recruited from 14-20 PHC centers located in socioeconomically disadvantaged areas in Stockholm County. The primary outcome will be measured in self-reported 7-day abstinence at 6 and 12 months after the intervention. The secondary outcomes will be measured in daily tobacco consumption, number of quit attempts, and health-related quality of life at 6 and 12 months after the intervention. Data will be collected through questionnaires and review of electronic medical records. Cost-effectiveness will be estimated through decision analytic modeling and measured by the incremental cost per quality-adjusted life year.

**Results:**

In the first set of PHC centers participating in the study, eight centers have been included. Recruitment of individual study participants is currently ongoing. Inclusion of a second set of PHC centers is ongoing with expected study start in September 2016.

**Conclusions:**

If TCP is found effective and cost-effective compared to standard treatment, the method could be implemented to facilitate tobacco cessation for socioeconomically disadvantaged groups in the PHC setting in Sweden.

**Trial Registration:**

International Standard Randomized Controlled Trial Number (ISRCTN): 11498135; http://www.isrctn.com/ISRCTN11498135 (Archived by WebCite at http://www.webcitation.org/6kTu6giYQ)

## Introduction

Smoking is a major risk factor for more than 60 different diseases, out of which cardiovascular diseases, lung diseases and cancers are the most common [1]. In Sweden, tobacco use is estimated to cause approximately 12,000 deaths and 100,000 new cases of tobacco-related diseases each year [2], corresponding to 8% of the total disease burden in the country [3]. In addition to the negative effects that tobacco use has on the health and quality of life of the population [4], it is also associated with increased costs for the health care system and for society at large [5]. Currently, 20% of the general adult population in Sweden are daily tobacco users [6]. However, the prevalence of tobacco use is unequally distributed as it is almost twice as high in lower socioeconomic groups compared to higher socioeconomic groups [7].

Since tobacco cessation has been found to reduce the risk of premature morbidity and mortality caused by tobacco-related diseases [8], it is a prioritized area in Swedish public health policy [9]. Treatment guidelines for tobacco cessation in the health care setting have been issued both on the national level in Sweden [1] and the regional level, eg, in Stockholm County [10]. The guidelines recommend that health care providers should offer cessation support to all daily smokers [1]. Although tobacco cessation interventions are one of the most cost-effective interventions available in health care [11], the treatment intensity for tobacco cessation is relatively low in Sweden [12]. In addition, high-risk groups that have an increased need of support are often not reached by such health promoting activities [1]. This could partly be explained by a lower motivation, self-efficacy and social support for quitting, a limited understanding of the harmful effects of tobacco use, and a stronger addiction to tobacco among tobacco users in lower compared to those in higher socioeconomic groups [13]. Other influencing factors include targeted marketing by the tobacco industry and lower adherence to treatment [13]. Moreover, there is a lack of awareness in this target group regarding available treatment options and misconceptions regarding the use and effectiveness of such services [14]. In addition, costs related to seeking and completing treatment for tobacco cessation present particular barriers for socioeconomically disadvantaged tobacco users [14,15]. The need for a more systematic approach and improved access to cessation support for socioeconomically disadvantaged groups has recently been emphasized [16]. There is also a need for more knowledge and training for PHC staff in how to communicate with and empower disadvantaged groups for efficient health promotion [17,18].

Studies conducted on health care consumption in Stockholm in different social groups show that individuals with foreign background, low educational level and low income visit primary health care (PHC) more often than their counterparts [19]. The public has confidence in the health care system [20] and most tobacco users seek care for different health problems at PHC centers. In addition, 87% of patients are positive towards receiving advice on lifestyle changes from health care providers [20]. According to the Swedish Healthcare Act, PHC has the main responsibility for health promotion and disease prevention in the Swedish health care system [21]. Therefore, PHC can be seen as a potential platform to improve the reach of health promoting activities, such as tobacco cessation support, to socioeconomically disadvantaged groups.

In a recent study, the perceived feasibility and optimal design of Tobacco Cessation on Prescription (TCP) as a PHC intervention targeting disadvantaged groups in Sweden, was explored [22]. The study found that TCP was perceived as a useful tool for tobacco users and health care providers and that it could facilitate a more structured and effective approach to tobacco cessation in the future compared to current tobacco cessation practices in PHC [22]. Based on these findings, there is now a hypothesis that TCP could be implemented and prescribed in a similar manner as Physical Activity on Prescription (PAP). PAP has been found effective in changing behavior and improving health and quality of life and is already in use in the PHC setting in Sweden to prevent disease and promote health in the general population [23]. A prescription approach to tobacco cessation could potentially increase the treatment intensity of tobacco cessation in the PHC setting and thus lead to decreased tobacco use and improved health in the target population. The aim of this study is to evaluate the effectiveness and cost-effectiveness of TCP as PHC intervention targeting socioeconomically disadvantaged groups in Stockholm, Sweden.

## Methods

### Study Design

**Figure 1:**
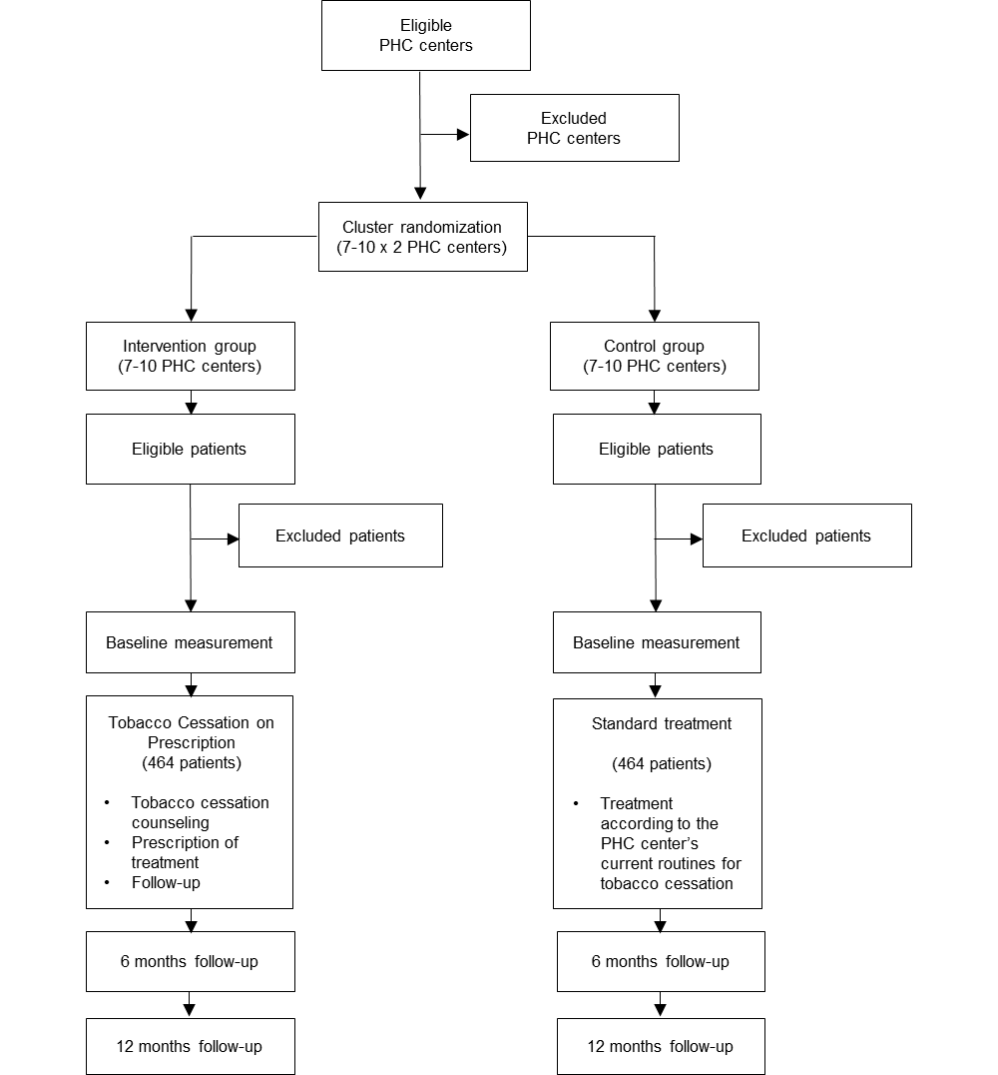
Flow chart of the study design.

In order to evaluate the effectiveness and cost-effectiveness of the intervention, a two-armed pragmatic cluster-randomized controlled trial [24], with an economic evaluation as a component, has been chosen as the study design. In total, 14-20 PHC centers will be randomized to either intervention or control conditions with a 1:1 ratio. Study participants in the control arm will be offered standard treatment, while study participants in the intervention arm will be offered TCP as a complement to current treatment practices for tobacco cessation at the PHC center. Measurement of patient outcomes will be conducted at baseline and 6 and 12 months after the intervention. The trial has been approved by the Regional Ethical Review Board in Stockholm (ref: 2015/207-31, 2015/1226-32). The study details in this protocol are presented according to the SPIRIT 2013 Statement to ensure high quality in reporting [25]. The study design is presented in Figure 1.

### Study Setting and Participants

Study participants will be recruited from participating PHC centers located in socioeconomically disadvantaged areas in Stockholm County, Sweden. Eligible PHC centers will be identified based on a socioeconomic index, which takes into account the income, educational level, ethnicity and health status of the population in a PHC center’s catchment area [26]. Daily tobacco users over 18 years of age with Swedish social security numbers and permanent residence permits, fluent in one of the two most common languages in the study setting, Swedish or Arabic, will be eligible for inclusion in the study. Daily tobacco use will be defined as daily use of cigarettes, snus (smokeless tobacco) or other tobacco products for at least the last year. Ongoing treatment for tobacco cessation and cognitive impairment affecting ability to participate in the study on a voluntary basis will be applied as exclusion criteria.

### Sampling and Recruitment

PHC centers located in areas with low socioeconomic status in Stockholm County will be identified through the previously mentioned socioeconomic index [26], purposively sampled by the researchers and invited to participate in the study. The managers at the PHC centers will be contacted via telephone by the researchers and offered further information via email and a physical meeting before agreeing to participate in the study.

Study participants will be recruited by one to three appointed providers employed at each of the participating PHC centers. However, all staff at the participating PHC centers will be able to refer patients to recruiting staff for more information about the study. In order to reduce selection bias, eligible participants will be identified through a short screening questionnaire before being invited to participate. Further information about the study will be administered by the recruiting staff at the participating PHC centers and written informed consent to participate sought before invited participants will be included in the study. Staff responsible for the recruitment of study participants will receive a brief training in the study design and recruitment procedure before the study start. Recruiting staff will also receive posters to help facilitate the recruitment of individual study participants. The recruitment period is expected to last for 18 to 24 months.

### Interventions

#### Tobacco Cessation on Prescription (Intervention)

**Figure 2:**
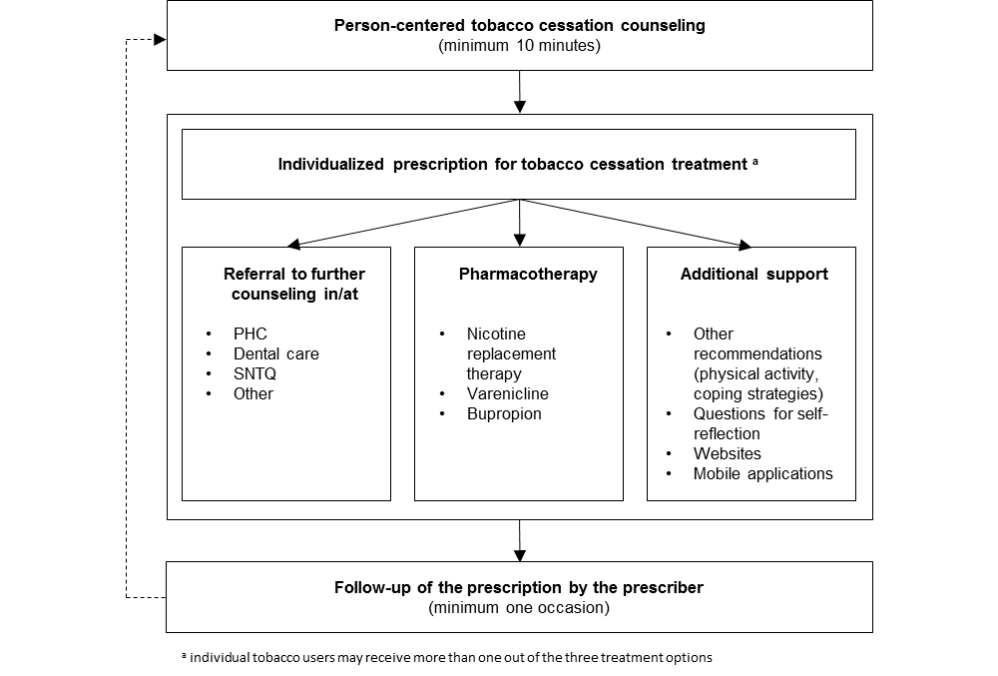
Conceptual model for TCP.

The TCP method is based on the PAP concept, which consists of person-centered counseling on physical activity, individualized prescription of physical activity, co-operation between prescribers and providers of physical activity, follow-up of the prescription, and a comprehensive manual that describes for which indications and how the method should be used [27]. In the TCP method, the components in PAP have been adjusted to the tobacco cessation context (see conceptual model in Figure 2 and full description of the core components below). The initial TCP prescription form was drafted by the researchers based on the national guidelines for tobacco cessation treatment in Sweden [1] and the results from the qualitative study that explored the perceived feasibility and optimal design of TCP [22]. The prescription form was then further developed based on an iterative process of feedback from waiting room interviews with patients, as well as workshops and written correspondence with health care providers, researchers and experts on tobacco cessation and lifestyle interventions already available by prescriptions in Sweden. The intervention design was also adjusted based on feedback from PHC providers that pilot tested the TCP method during a 6-week period at a PHC center located in a socioeconomically disadvantaged area in Stockholm.

Prior to the administration of the intervention, one to three PHC providers per center, responsible for the treatment of patients in the intervention group, will receive 4 hours of training by representatives from the Swedish National Tobacco Quitline (SNTQ) [28] and the Stockholm County Council (the regional authority and health care provider) in available treatment options for tobacco cessation and the TCP method. A manual which summarizes the training and describes how the prescription form should be filled out and how it can be used in tobacco cessation counseling, has been developed and will be distributed in connection with the education of PHC providers in the intervention group.

The core components of the intervention consist of tobacco cessation counseling of tobacco users according to the TCP method. This is defined as tobacco cessation counseling (minimum 10 minutes) provided by a qualified health care professional in combination with a prescription for individualized tobacco cessation treatment, including options for (1) further counseling (referral to a health care provider with more competence or SNTQ), (2) pharmacotherapy (nicotine replacement therapy, varenicline, bupropion), (3) other measures for tobacco cessation (physical activity and other strategies to cope with withdrawal symptoms), (4) follow-up (by telephone or revisit) and (5) support for self-management (questions for self-reflection, reference to mobile applications, Web-based counseling and websites for more information and support). The approach will be individualized in the sense that providers will discuss the available treatment options, contraindications, preferences, and other relevant circumstances with the patient and then decide together which treatment alternative(s) suit the individual best. The TCP method also includes follow-up of the prescription by the prescriber on at least one occasion.

#### Standard Treatment (Control)

Standard treatment is defined as treatment for tobacco cessation according to current practices at the PHC center. Since the choice of tobacco cessation treatment varies depending on individual characteristics and preferences of tobacco users and it is up to each PHC center to decide for themselves how their tobacco cessation services should be organized, the treatment components (eg, type of counseling and pharmacotherapy) are expected to vary both within and between the study arms. Thus, the provided treatments will be documented by one to three PHC providers per center in the control group responsible for the treatment of study participants and further defined retrospectively. Since the same treatment components are likely to be present in both study arms, it is important to state that the major difference between them is how the counseling is administered (with or without a prescription form). The minimum intervention for the control group is a brief advice (<5 minutes).

To ensure that the difference between the trial conditions is dependent on the prescription form and not on the training of PHC staff, the PHC providers responsible for the treatment of patients in the control group will receive 3.5 hours of training by representatives from SNTQ and the County Council in available treatment options for tobacco cessation prior to the study start (the same training as the intervention group, excluding the 30 minute TCP component). A manual identical to the one developed for the intervention group, excluding all information about the TCP method and summarizing the training, has been developed and will be distributed in connection with the education of PHC providers in the control group.

### Outcomes

The primary outcome of the intervention will be measured in self-reported point prevalence of 7-day abstinence (total abstinence from tobacco use during the 7 days preceding follow-up) at 6 months after the intervention. The secondary outcomes are self-reported point prevalence of 7-day abstinence at 12 months after the intervention and 3-month continued abstinence, daily tobacco consumption (number of cigarettes), number of quit attempts (periods of total abstinence from tobacco use for more than 24 hours) and health-related quality of life (on a scale from 0-1 where 0 represents death and 1 represents perfect health) at 6 and 12 months after the intervention. All outcomes will be based on patients' self-reports. Cost-effectiveness will be measured as the incremental cost per quality-adjusted life year.

### Data Collection

Data on sociodemographic characteristics, tobacco use, and nicotine dependence, previous quit attempts, self-efficacy and motivation to quit, health status and health-related quality of life will be collected through patient questionnaires. The questionnaires are based on questions from the Swedish Public Health Survey 2014 [29], a questionnaire that was used in a previous study that evaluated the effectiveness of brief advice for tobacco cessation in dental practices in Sweden [30] and the Swedish and Arabic (Lebanon) version of the EQ-5D-5L instrument [31]. Questions not included in the EQ-5D-5L questionnaire were translated from Swedish to Arabic and back by two different professional translation agencies and critically reviewed by a research assistant fluent in both Swedish and Arabic. The questionnaires were pilot tested in Swedish prior to the study start and in Arabic prior to the recruitment of Arabic speaking participants.

The measurements will be conducted at baseline (before the intervention) and 6 and 12 months after the intervention. In the follow-up questionnaires, questions regarding the tobacco cessation-related care the patients have received during the study period have been added. The baseline questionnaires will be administered by staff responsible for the treatment of patients at the participating PHC centers. The PHC providers administering the treatment will also document what treatment the patients have received (duration, content, intensity and number of visits, mode of counseling, referrals, any recommended and prescribed pharmacotherapy, follow-ups, etc) in the electronic medical records and in study specific documentation protocols. Staff will be educated in the documentation procedures before the start of the study. The follow-up questionnaires will be sent to the participants via mail by the researchers to avoid attrition caused by additional costs and administrative burden of revisits for the study participants. A reminder with a new follow-up questionnaire attached will be sent out via mail by the researchers if the follow-up questionnaire is not returned within ten days. If the reminder questionnaire is not returned, additional reminders to return the questionnaire will be sent out via mail, email and SMS text messaging (short message service, SMS) by the researchers. In connection with the second reminder, the participants will be offered the opportunity to answer the questionnaire in a telephone interview. Multiple reminders and forms of contact have been found essential in promoting high retention rates among disadvantaged research participants who are often highly mobile [32]. The strategy above is expected to be sufficient in reaching study participants who are willing to respond to the follow-up questionnaires. Arabic speaking staff will assist the researchers in contacting and collecting data from the Arabic speaking participants.

Additional data on the characteristics of participating PHC centers and providers delivering the intervention will be collected through questionnaires and interviews. This includes data on organizational aspects such as number of listed patients, number of employees, and type of professions and routines for tobacco cessation at the PHC center level and data on age, sex, profession, qualifications, and personal experiences of tobacco use and tobacco cessation at the PHC provider level. Data on structural changes in the PHC centers during the study period (eg, staff turnover) will also be collected retrospectively. This data will be collected by the researchers.

### Sample Size

The required sample size was calculated based on the primary outcome, assuming a 7% rate of 7-day prevalence in abstinence from tobacco use at 6 months follow-up in the control group, significance level of 5% and 80% power to detect a 2.0 relative risk of successful quit attempts in the intervention group compared to the control group. The estimated prevalence of abstinence in the control group corresponds to the success rate of brief advice [33] which is the minimum intervention for the control group in this study. The estimated relative risk corresponds to the relative risk of abstinence from tobacco use for combined pharmacotherapy and counseling, as recommended for the intervention group in this study, compared to minimal intervention or usual care [34]. The unadjusted sample size was found to be 300 per arm. Assuming an intra-cluster coefficient (ICC) of 0.01 according to Adams et al [35] the sample size was adjusted for design effect using the formula: 1 + (m − 1) ρ, where m represents the mean number of participants in each cluster and ρ is the ICC. Having 7 clusters per arm and 43 participants per cluster, the adjusted sample size will be 426 per arm. Finally adjusted for an attrition/drop-out of 8%, the final sample size will be 464 per arm or 928 in total. The estimated attrition rate of 8% is based on reported attrition rates from two other tobacco cessation trials with similar study designs, both conducted in the Swedish health care setting [30,36]. A higher number of participating PHC centers could decrease the required number of study participants without compromising the power to detect a statistically significant difference in the outcomes between the groups at follow-up, wherefore the aim is to recruit a total of 20 PHC centers. A total sample size of 840 would then be needed.

### Randomization

A computer generated random allocation sequence will be applied to randomize the PHC centers to either intervention or control conditions with a 1:1 ratio. Cluster-randomization will be employed at the PHC center level, meaning that all individual study participants recruited from a particular PHC center will receive the same treatment. This will be done due to feasibility reasons and to avoid contamination of the trial conditions. The PHC centers will be paired based on their socioeconomic index and allocated to the treatment conditions from each pair after they have agreed to participate, approximately one month before the PHC provider training. Each set of participating PHC centers will be randomized separately. The randomizations will be conducted by a statistician.

The PHC providers and study participants will not be blinded. However, the study participants will not be informed about the difference between the trial conditions until after the study. This will be done in order to avoid attrition and preconceptions regarding the treatment effectiveness that could affect the study results (risk that study participants in the control group could perceive standard treatment as less effective compared to TCP).

### Statistical Analysis

In order to describe the setting and the effectiveness of the randomization, descriptive statistics of the study population’s baseline characteristics at both individual and cluster level will be presented separately for the intervention and control arm, as proportions for categorical variables and as mean values with corresponding standard deviation (SD) for continuous variables.

The association between the treatment and the outcomes post-intervention will be analyzed using multiple regression models. A logistic regression model will be used for binary outcomes, including the primary outcome, 7-day abstinence. The result will be presented as an odds ratio (OR) and corresponding 95% confidence interval (CI). The association between the treatment and continuous/count outcomes, including the secondary outcomes, daily tobacco consumption, number of quit attempts and health-related quality of life, will be analyzed using multiple linear and Poisson regression models. All analyses will be conducted according to the intention to treat principle [37], meaning that the individual study participants will be analyzed according to how the PHC centers where they were recruited and treated were randomized, regardless of which intervention they received. Inference will be targeted at the individual level and hierarchical models will be used to handle potential clustering on the PHC center level. Model covariates will include age, sex, educational level, nicotine dependence, motivation and readiness to quit, previous quit attempts, previous use of pharmacotherapy, and diagnosis of chronic disease. For main analysis no missing data will be imputed. However, classical multiple imputation methods will be used for an additional sensitivity analysis if any of the included variables have more than 5% missing observations. The analyses will be conducted by the research team, including a statistician.

### Process Evaluation

A process evaluation will be conducted to measure implementation outcomes such as service delivery of tobacco cessation at the PHC center level and self-reported fidelity to the intervention at the PHC provider level and the participant level [38]. Data will be collected through review of electronic medical records, PHC provider documentation protocols and patient questionnaires. Semi-structured interviews with PHC providers and tobacco users will also be conducted and qualitatively analyzed with content analysis to explore the acceptability, appropriateness, adoption [38], and general experiences of TCP.

### Economic Evaluation

A health economic evaluation will be conducted alongside the trial to evaluate the cost-effectiveness of the intervention compared to standard treatment. This will be done by incorporating the trial results on effectiveness and data from other sources into a decision analytic model specifically developed to estimate the future costs and outcomes of tobacco cessation interventions. Decision analytic models are often used in health economic evaluations since they allow for synthesis of data from different sources and extrapolation of events beyond a clinical trial [39]. The analysis will be conducted from a societal perspective with a lifetime horizon where the incremental cost-effectiveness ratio (difference in cost, divided by the difference in effectiveness between the treatment alternatives), is defined as the additional cost in Swedish Krona (SEK) per quality-adjusted life year. Cost (resource use) and epidemiological data will be collected from the trial as well as from registers, reports, and previously published scientific articles. Cost data will include indirect costs of production loss and direct health care costs of PHC staff, pharmacotherapy, and other resources used in the delivery of tobacco cessation treatments and overhead costs in both study arms throughout the entire study period. Future health care costs will be calculated as average annual costs of health states included in the evaluation. Epidemiological data will include population data on life expectancy and relative risk of tobacco related diseases among tobacco users and former tobacco users. The evaluation, including discounting, sensitivity analysis, and reporting will be conducted based on best practice guidelines for health economic evaluations in Sweden [40].

## Results

From April to November 2015, eight PHC centers were recruited and randomly assigned to the trial conditions. The PHC providers responsible for the treatment of study participants were trained in February and April 2016. Recruitment of individual study participants is currently ongoing. Recruitment of a second set of PHC centers is also ongoing. The expected study start of the second set of PHC centers is in September 2016.

## Discussion

This study aims to evaluate the effectiveness and cost-effectiveness of a novel intervention that builds on previous research and experiences of prescribed lifestyle interventions in the PHC setting in Sweden that could potentially facilitate a more structured approach to tobacco cessation for socioeconomically disadvantaged groups compared to current practice. The method is based on clinical guidelines for tobacco cessation treatment in the Swedish health care setting [1] and has been developed in close collaboration with a variety of relevant stakeholders including the target population [32]. Stakeholders that have been involved in this process include researchers and experts on tobacco cessation and lifestyle interventions on prescription in Sweden (researchers experienced in intervention research, tobacco control and PAP, as well as representatives from the Swedish Professional Agency Against Tobacco and SNTQ), PHC providers (mainly nurses and physicians but also dieticians and occupational therapists) and tobacco users of various ages and sexes from PHC centers located in socioeconomically disadvantaged areas in Stockholm. Since community involvement in intervention design has been found to have positive effects on the uptake of an intervention [32], this is expected to have a positive effect on the effectiveness and acceptability of TCP. Results from the previously mentioned study that explored the perceived feasibility and optimal design of TCP suggest that there is support for the method [22].

A key concern when conducting the study is reaching the intended target population. For example, language barriers may limit the access to the most disadvantaged groups. However, the two most common languages in the target population, Swedish and Arabic, are considered in the study. It is important that the participating PHC centers have access to interpretation services, or staff fluent in these languages, and that the materials are available in both languages to enable recruitment of participants and delivery of the intervention as intended [32]. It is also important to consider that not all tobacco users who visit PHC centers located in socioeconomically disadvantaged areas have a low socioeconomic status. However, recruitment of patients to health interventions has been found much more effective in the PHC setting in socioeconomically disadvantaged areas compared to community approaches [41]. For feasibility and ethical reasons, a common research approach is to focus on socioeconomically disadvantaged areas rather than individuals when recruiting such populations. Since the intended target population may be difficult for outsiders of the community to reach [32], PHC staff will be responsible for recruitment of individual study participants. As in other studies conducted on disadvantaged populations, the participants will receive a gift certificate worth 100 SEK to promote their partaking in the research and increase retention rates [32]. In order to describe the study population and assess whether it is representative for the intended target population, data on socioeconomic status will be collected on the individual level at baseline.

A possible limitation of the study is due to self-reported tobacco-related outcomes. The accuracy of self-reported tobacco use tends to be lower compared to biochemical markers such as cotinine measurements and may lead to underestimates of tobacco use due to underreporting as a consequence of social desirability [42]. However, self-reported tobacco use is a common research approach and was chosen in this study due to budget restrictions and feasibility reasons. In addition to higher costs for equipment and training, cotinine measurement would require two more compulsory revisits per study participant which could compromise the retention rate due to an increase in administrative burden and costs for the study participants who have to pay out-of-pocket for their visits. This is expected to have a particularly negative impact on the retention of the participants in this study as they are recruited from socioeconomically disadvantaged areas. Cotinine measurements are also expected to decrease the willingness among PHC to participate due to the increased administrative burden of additional compulsory revisits. However, a correction factor may be used to adjust for underreporting which is expected to be lower than 10% [42].

Another potential limitation is that the education of PHC providers is relatively brief (4 hours). However, data collected at the PHC center level prior to the study start showed that the majority of the PHC providers responsible for the treatment of patients had previous training in tobacco cessation treatment, motivational interviewing or lifestyle counseling. Given this fact, the length of the education was considered sufficient by the participating PHC centers and the representatives from SNTQ and the County Council that were involved in designing the training of the PHC providers in the study.

A major strength of the study is the robustness of its design. The pragmatic approach will provide high external validity under real world conditions in the context under study [24] and lead to useful results for policy making and health systems development. Furthermore, the inclusion of data on cost-effectiveness will facilitate policy decisions on wider use of the program [24,39]. Another strength of the study is that it focuses on socioeconomically disadvantaged groups who have a greater need for tobacco cessation support due to the higher prevalence of tobacco use and difficulties in reaching this target group with health promoting interventions compared to groups with higher socioeconomic status. To the authors’ knowledge, this is the first study to evaluate the effectiveness and cost-effectiveness of tobacco cessation services targeting socioeconomically disadvantaged groups in the PHC setting in Sweden. The study is expected to offer valuable insights regarding how such services are currently organized. If TCP is proven to be effective and cost-effective, it will be a valuable tool for tobacco prevention that can be readily implemented to promote health among socioeconomically disadvantaged populations in the PHC setting in Sweden.

## Abbreviations

PAP: Physical Activity on Prescription
PHC: primary health care
SNTQ: Swedish National Tobacco Quitline
TCP: Tobacco Cessation on Prescription

## References

[1] National Board of Health and Welfare (2011). [National guidelines for disease prevention methods 2011. Tobacco use, alcohol consumption, physical inactivity and unhealthy dietary habits. Support for control and management].

[2] National Board of Health and Welfare (2014). [Register data on the harmful effects of tobacco use].

[3] Agardh E, Boman U, Allebeck P (2015). [Alcohol, drugs and tobacco smoking causes much of the burden of disease--Trends in Sweden 1990-2010 mapped based DALY method]. Lakartidningen.

[4] Lyons RA, Lo SV, Littlepage B (1994). Perception of Health amongst ever-smokers and never-smokers: a comparison using the SF-36 Health Survey Questionnaire. Tobacco Control.

[5] Bolin K, Borgman B, Gip C, Wilson K (2011). Current and future avoidable cost of smoking--estimates for Sweden 2007. Health Policy.

[6] Public Health Agency of Sweden (2015). [Tobacco use - national results and time series 2014 (Excel-document, 1,3 MB)].

[7] Galanti M, Gilljam H, Post A, Eriksson B (2011). [Tobacco use in the county].

[8] U.S. Department of Health and Human Services (2014). The health consequences of smoking - 50 years of progress A report of the Surgeon General.

[9] Reinfeldt F, Larsson M (2007). [Government proposition 2007/08:110 A renewd public health policy].

[10] Stockholm County Council (2015). [Regional health care program for health promoting lifestyle habits].

[11] Ruger JP, Lazar CM (2012). Economic evaluation of pharmaco- and behavioral therapies for smoking cessation: a critical and systematic review of empirical research. Annu Rev Public Health.

[12] Swedish Institute of Public Health (2010). [On the way to a tobacco-free County Council - Monitoring of County Council and regional policy work on tobacco prevention 2009].

[13] Hiscock R, Bauld L, Amos A, Fidler J, Munafò Marcus (2012). Socioeconomic status and smoking: a review. Ann N Y Acad Sci.

[14] Roddy E, Antoniak M, Britton J, Molyneux A, Lewis S (2006). Barriers and motivators to gaining access to smoking cessation services amongst deprived smokers--a qualitative study. BMC Health Serv Res.

[15] Bonevski B, Bryant J, Paul C (2011). Encouraging smoking cessation among disadvantaged groups: a qualitative study of the financial aspects of cessation. Drug Alcohol Rev.

[16] Regional cancer centers in collaboration (2014). [RCC:s action plan for a smoke-free Sweden].

[17] National Board of Health and Welfare (2011). [Unequal conditions for health and health care - equality perspectives on health care].

[18] Tomson T, Tomson G, Savage C (2012). [The educational system of today and health personnel of tomorrow]. Lakartidningen.

[19] Walander A, Ålander S, Burström B (2004). [Social differences in health care utilization].

[20] Swedish Association of Local Authorities and Regions (2015). [The health care barometer 2014].

[21] (1982). Healthcare act (1982:763 5 §).

[22] Leppänen A, Biermann O, Sundberg CJ, Tomson T (2016). Perceived feasibility of a primary care intervention for Tobacco Cessation on Prescription targeting disadvantaged groups in Sweden: a qualitative study. BMC Res Notes.

[23] Kallings L (2008). Physical activity on prescription: Studies on physical activity level, adherence and cardiovascular risk factors. PhD thesis.

[24] Patsopoulos NA (2011). A pragmatic view on pragmatic trials. Dialogues Clin Neurosci.

[25] Chan A, Tetzlaff J, Altman D (2013). SPIRIT 2013 Statement: Defining Standard Protocol Items for Clinical Trials. Ann Intern Med.

[26] Burström B, Walander A, Viberg I, Bruce D, Agerholm J, Ponce de Leon A (2013). [Proposal for socioeconomic index 2011-2013].

[27] Swedish Institute of Public Health (2011). [PAP - Individual based prescription of physical activity].

[28] Nohlert E, Ohrvik J, Helgason ÁR (2014). Effectiveness of proactive and reactive services at the Swedish National Tobacco Quitline in a randomized trial. Tob Induc Dis.

[29] Public Health Agency of Sweden (2014). [Aim and background of the questions in the national public health survey].

[30] Virtanen SE, Zeebari Z, Rohyo I, Galanti MR (2015). Evaluation of a brief counseling for tobacco cessation in dental clinics among Swedish smokers and snus users. A cluster randomized controlled trial (the FRITT study). Preventive Medicine.

[31] Janssen MF, Pickard AS, Golicki D, Gudex C, Niewada M, Scalone L, Swinburn P, Busschbach J (2012). Measurement properties of the EQ-5D-5L compared to the EQ-5D-3L across eight patient groups: a multi-country study. Qual Life Res.

[32] Bonevski B, Randell M, Paul C, Chapman K, Twyman L, Bryant J, Brozek I, Hughes C (2014). Reaching the hard-to-reach: a systematic review of strategies for improving health and medical research with socially disadvantaged groups. BMC Med Res Methodol.

[33] Aveyard P, Begh R, Parsons A, West R (2012). Brief opportunistic smoking cessation interventions: a systematic review and meta-analysis to compare advice to quit and offer of assistance. Addiction.

[34] Stead L, Lancaster Tim (2012). Combined pharmacotherapy and behavioural interventions for smoking cessation. Cochrane Database Syst Rev.

[35] Adams G, Gulliford MC, Ukoumunne OC, Eldridge S, Chinn S, Campbell MJ (2004). Patterns of intra-cluster correlation from primary care research to inform study design and analysis. Journal of Clinical Epidemiology.

[36] Nohlert E, Tegelberg Å, Tillgren P, Johansson P, Rosenblad A, Helgason ÁR (2009). Comparison of a high and a low intensity smoking cessation intervention in a dentistry setting in Sweden – a randomized trial. BMC Public Health.

[37] Campbell M, Machin D, Walters S (2007). Medical statistics: a textbook for the health sciences.

[38] Proctor E, Silmere H, Raghavan R, Hovmand P, Aarons G, Bunger A, Griffey R, Hensley M (2010). Outcomes for Implementation Research: Conceptual Distinctions, Measurement Challenges, and Research Agenda. Adm Policy Ment Health.

[39] Drummond M, Sculpher M, Torrance G, O'Brien B, Stoddart G (2005). Methods for the economic evaluation of health care programmes.

[40] The Dental and Pharmaceutical Benefits Agency [General advice on economic evaluations].

[41] Thompson TP, Greaves CJ, Ayres R, Aveyard P, Warren FC, Byng R, Taylor RS, Campbell JL, Ussher M, Michie S, West R, Taylor AH (2015). Lessons learned from recruiting socioeconomically disadvantaged smokers into a pilot randomized controlled trial to explore the role of Exercise Assisted Reduction then Stop (EARS) smoking. Trials.

[42] Connor Gorber S, Schofield-Hurwitz S, Hardt J, Levasseur G, Tremblay M (2009). The accuracy of self-reported smoking: a systematic review of the relationship between self-reported and cotinine-assessed smoking status. Nicotine Tob Res.

